# Prognosis in Hepatic Cirrhosis

**Published:** 1907-05-04

**Authors:** 


### Clinical Points.

### PROGNOSIS IN HEPATIC CIRRHOSIS.

When patients present themselves with definite symptoms of cirrhosis of the liver, one of the questions which arise is, How long is this patient likely to live? When there is ascites, the answer is easy: The patient is not likely to live much more than a year, and he may very probably die sooner. When the main symptom is jaundice without ascites, the prognosis is more open; the patient may die quickly of cholæmia, or he may recover from the jaundice and live for many years before the ascitic stage is reached. When the main symptom is hæmatemesis without either jaundice or ascites, it is unusual for the patient not to recover for the time being, and to go on living for a considerable time afterwards.

The following is an interesting example of this. The case teaches three things. First, that a patient may survive for a great number of years after the cirrhotic changes have begun; secondly, that after indulging in alcohol a patient may eschew it entirely and yet die of cirrhosis of the liver, even after being absolutely teetotal for seventeen years; and, thirdly, that ascites in association with cirrhosis is evidence that the end is near.

The patient, a respectable, white-bearded, elderly gentleman of 72 years of age, had all his life been employed by the same firm of wine-merchants, who had extensive cellars of the best wines. Up till seventeen years previously he had been in the habit of tasting the various wines frequently all day as part of his trade. He had never been genuinely intoxicated, but he acknowledged that he had often and often had occasion to take "quite as much as was good for him." When he was 55 years old he had an illness, during which he vomited blood, and his doctor urged him to give up all alcoholic drinks entirely. This he did. He recovered, and remained perfectly well, so far as he could judge, until, when 72, his abdomen began to swell. He had not touched a drop of alcohol during the whole of these seventeen years, but it seemed obvious that the ascites was due to cirrhosis of the liver—a diagnosis which was confirmed by autopsy four months later. Paracentesis abdominis was performed twice before he died; the liver was a typical contracted hobnailed organ. It is possible, of course, that the alcoholism of his earlier life had only initiated changes which ultimately gave rise to the cirrhosis; but it seems more likely, in view of the hæmatemesis when he was 55, that cirrhosis had already begun then, and may even have been considerable; and that he either lived in spite of a full degree of cirrhosis for 17 years, and that only as he grew older did the weakest organ in his body, his liver, give way first; or else that, in spite of becoming teetotal, the fibrosis of the liver, already existent when he was 55, slowly progressed, until it ultimately killed him. It is at any rate interesting to know that a patient who was formerly an alcoholic, but who has been teetotal for years, afterwards cannot be guaranteed free from the dangers of cirrhosis of the liver.

